# The Iron-Inflammation Axis in Early-Stage Triple-Negative Breast Cancer

**DOI:** 10.3389/fcell.2022.784179

**Published:** 2022-02-23

**Authors:** Fangfang Duan, Muyi Zhong, Jinhui Ye, Li Wang, Chang Jiang, Zhongyu Yuan, Xiwen Bi, Jiajia Huang

**Affiliations:** ^1^ Department of Medical Oncology, The State Key Laboratory of Oncology in South China, Collaborative Innovation Center for Cancer Medicine, Sun Yat-sen University Cancer Center, Guangzhou, China; ^2^ Department of Breast Oncology, Dongguan People’s Hospital, Dongguan, China; ^3^ Department of Breast Oncology, The First People’s Hospital of Zhaoqing, Zhaoqing, China

**Keywords:** early-stage triple-negative breast cancer, serum iron level, monocyte-tolymphocyte ratio, predictive nomogram, survival

## Abstract

The iron-related homeostasis and inflammatory biomarker have been identified as prognostic factors for cancers. We aimed to explore the prognostic value of a novel comprehensive biomarker, the iron-monocyte-to-lymphocyte ratio (IronMLR) score, in patients with early-stage triple-negative breast cancer (TNBC) in this study. We retrospectively analysed a total of 257 early-stage TNBC patients treated at Sun Yat-sen University Cancer Center (SYSUCC) between March 2006 and October 2016. Their clinicopathological information and haematological data tested within 1 week of the diagnosis were collected. According to the IronMLR score cutoff value of 6.07 μmol/L determined by maximally selected rank statistics, patients were stratified into the low- and high-IronMLR groups, after a median follow-up of 92.3 months (95% confidence interval [CI] 76.0–119.3 months), significant differences in 5-years disease-free survival (DFS) rate (81.2%, 95% CI 76.2%–86.5% *vs.* 65.5%, 95% CI 50.3%–85.3%, *p* = 0.012) and 5-years overall survival (OS) rate (86.0%, 95% CI 81.6%–90.7% *vs.* 65.5%, 95% CI 50.3%–85.3%, *p* = 0.011) were seen between two groups. Further multivariate Cox regression analysis revealed the IronMLR score as an independent predictor for DFS and OS, respectively, we then established a prognostic nomogram integrating the IronMLR score, T stage and N stage for individualized survival predictions. The prognostic model showed good predictive performance with a C-index of DFS 0.725 (95% CI 0.662–0.788) and OS 0.758 (95% CI 0.689–0.826), respectively. Besides, calibration curves for 1-, 3-, 5-DFS, and OS represented satisfactory consistency between actual and nomogram predicted survival. In conclusion, the Iron-inflammation axis might be a potential prognostic biomarker of survival outcomes for patients with early-stage TNBC, prognostic nomograms based on it with good predictive performance might improve individualized survival predictions.

## Introduction

Triple-negative breast cancer (TNBC), characterized by the absence of estrogen receptor (ER), progesterone receptor (PR), and human epidermal growth factor receptor 2 (HER-2) expression, accounts for approximately 15% of all breast cancers (Ferlay et al., 2021; Wang et al., 2021). Compared to other subtypes of breast cancers, patients with TNBC usually experience worse survival outcomes due to its increased aggressiveness, heterogeneity, and risks of recurrence and metastasis (Li et al., 2017). Over the past few decades, despite great therapeutic advances in TNBC, TNBC remains a considerable challenge for women worldwide due to its relatively high mortality (Dent et al., 2007; Bianchini et al., 2016; Garrido-Castro et al., 2019). Therefore, the discovery of novel, precise biomarkers, or individualized therapeutic targets for patients with TNBC is urgently needed.

As an essential trace element, iron is involved in activating many proteins, enzymes, and biological processes, such as cell respiration and various signalling pathways (Torti et al., 2018). Increasing studies have demonstrated that the dysregulation of systemic iron homeostasis is related to tumor initiation, growth, development, and progression (Chen et al., 2015; Radulescu et al., 2016). The depletion of iron using iron chelators or targeting increased serum iron has been explored as a novel therapeutic strategy for some cancers (Nutting et al., 2009; Yamasaki et al., 2011; List et al., 2012; Neufeld et al., 2012; Kalinowski et al., 2016; Stockwell et al., 2017; von Hagens et al., 2017). Moreover, ferroptosis, an iron-dependent programmed cell death, has been classified as a type of regulated necrosis and is also recognized as a new therapeutic target for tumours (Dixon et al., 2012). Given its immunogenicity, ferroptosis can induce cells to release damage-associated molecular patterns (DAMPs) and alarmins, which might enhance cell death and facilitate a series of inflammation-related responses (Martin-Sanchez et al., 2017; Proneth and Conrad, 2019; Sun et al., 2020). However, the role of ferroptosis in inflammation is multifaceted, it not only induces inflammatory activity but also suppresses inflammatory cell infiltration (Umemura et al., 2017; Tsurusaki et al., 2019; Sun et al., 2020; Zhou et al., 2020).

Monocytes have been explored for the suppression of lymphocyte activation, which is associated with aggressive tumors and metastasis (Tiainen et al., 2021). Peripheral lymphocytes or lymphocytes infiltrating in the tumour microenvironment (TME) play an important role in antitumour immunity by T cell-mediated cellular cytotoxicity (Andre et al., 2013). Therefore, the serum monocyte-to-lymphocyte ratio (MLR) might serve as a predictor of systemic inflammatory status, and an increased MLR might reflect poor antitumour immunity (Miklikova et al., 2020). The prognostic value of the MLR has been previously explored in breast cancer (De Giorgi et al., 2019). Thus, we hypothesized that a comprehensive biomarker based on iron homeostasis and systemic inflammation status might also show potential prognostic value. However, to date, no study has examined this aspect.

We aimed to explore the prognostic value of the novel comprehensive biomarker, the IronMLR score, calculated based on the serum iron level and MLR, for female patients with early-stage TNBC. Subsequently, we attempted to establish a prognostic model based on this Iron-inflammation axis for individualized survival predictions.

## Methods

### Eligible Patients

We explored the predictive value of a novel biomarker, the IronMLR score, in female patients newly diagnosed with early-stage TNBC at Sun Yat-sen University Cancer Center (SYSUCC) between March 2006 and October 2016. Our study protocol was approved by the Ethics Committee of SYSUCC (registration number: B2021-282-01). Given the retrospective nature of the current study, the requirement of written informed consent from patients was waived. In addition, we anonymously analysed all personal data in line with the Declaration of Helsinki.

Patients were included if they met the following key inclusion criteria: 1) 18 ≤ age <75 years old; 2) pathological diagnosis of breast cancer; 3) absence of ER, PR, and HER2 expression (scored as 0, 1+, or 2+ by immunohistochemistry [IHC] without amplification of the ERBB2 gene on fluorescence *in situ* hybridization); 4) no local relapse or distant metastasis at the diagnosis; and 5) complete clinicopathological data and available haematological parameters assessed within 1 week of the date of diagnosis. All patients in this study were restaged according to the American Joint Committee on Cancer (AJCC 2010, seventh version).

Key exclusion criteria included 1) pregnancy or lactation; 2) a history of malignancy, including breast cancer; 3) medication affecting the patient’s inflammatory status before diagnosis; and 4) the presence of any severe or uncontrolled complications.

### Information Collection and Measurement of Serum Iron Levels

Clinicopathological data were hand-retrieved from the electronic medical records system of our hospital and included age, menstrual status, histological type, T stage, N stage, histological grade, lymphovascular invasion, and Ki-67 index. Haematological parameters were assessed within 1 week of initiating any anti-tumor therapy, and the MLR was calculated as: MLR = monocyte count (10^9^/L)/lymphocyte count (10^9^/L). We acquired patient blood samples obtained within 1 week of diagnosis from the Tumor Resource Library of SYSUCC. The serum iron levels of patients were analysed using the Iron (Fe) Assay Kit (PAESA Chromogenic Method) with a Cobas 8,000 system (Roche Diagnostics, Basel, Switzerland). The IronMLR score was calculated as following: IronMLR score = serum iron level * MLR.

### Follow-up and Endpoints

We retrieved patient follow-up information from the outpatient electronic records of our center or telephone interviews. Patients were monitored every 3 months during 2 years of the diagnosis, subsequently every 6 months to 5 years, and then once every year thereafter. The main follow-up items included routine laboratory tests, menstrual status, ultrasound (breast and abdomen), or computed tomography (CT). Patients underwent bone scintigraphy annually.

The primary endpoint of the current study was disease-free survival (DFS), which was defined as the time from the date of diagnosis to the date of disease progression or death due to any cause. The second endpoint was overall survival (OS), which was defined as the time from the date of diagnosis to the date of death due to any cause.

### Statistical Analysis

Categorical variables are presented as frequencies with percentages, and continuous variables are listed as medians with interquartile ranges (IQRs). Chi-square tests and Mann-Whitney U tests were performed to compare the association between patient clinicopathological characteristics and the baseline IronMLR score. Patients with early-stage TNBC were stratified into high and low IronMLR groups according to the cut-off value of the IronMLR score determined by maximally selected rank statistics. The Kaplan-Meier method was performed to estimate survival curves of two different IronMLR score groups, and the log-rank test was used to compare their survival differences. Only variables with *p* values less than 0.05 in the univariate Cox analysis were included in the multivariate Cox regression model. Factors were tested according to the Schoenfeld residuals (Wileyto et al., 2013), and their corresponding hazard ratios (HRs) with 95% confidence intervals (CIs) were estimated. Then, we developed a prognostic model integrating the IronMLR score with other independent clinicopathological indicators from the multivariate Cox analysis, and we evaluated the prognostic accuracy and discriminative ability of the predictive model by means of the concordance index (C-index), calibration curves, and time-dependent receiver operating characteristic (ROC) curves. A two-sided *p <* 0.05 was regarded as significant, and all statistical analyses were performed using R 4.0.1.

## Results

### Patient Clinicopathologic Characteristics According to the IronMLR Score

**FIGURE 1:**
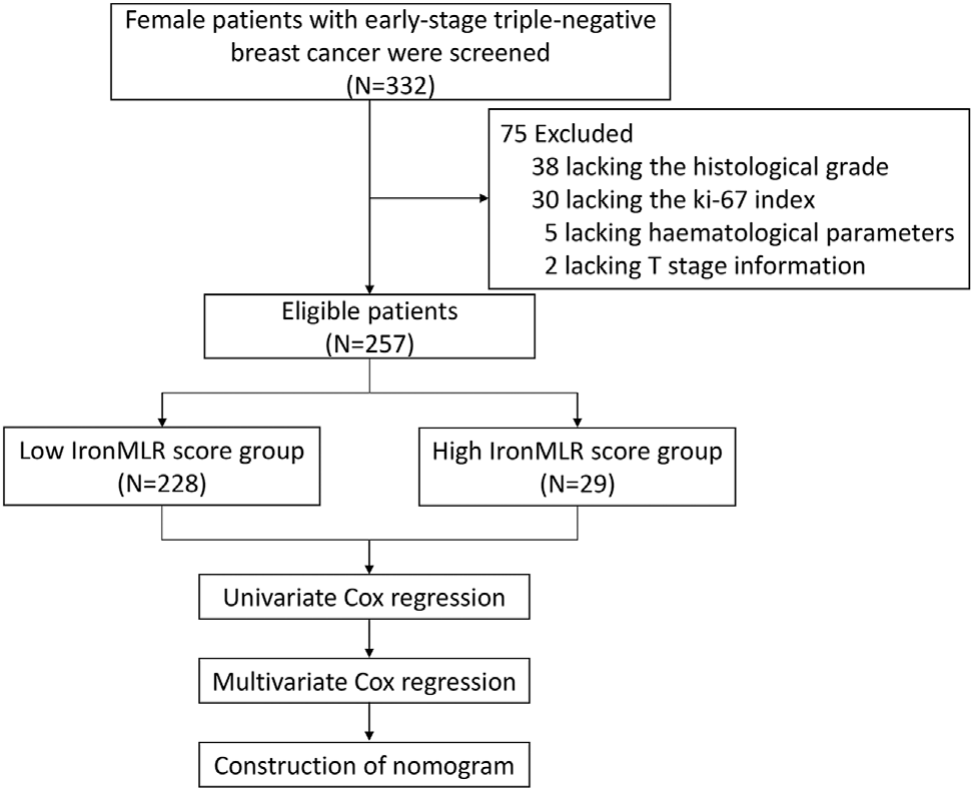
Flow chart of patients selection in this study.

As presented in the flow chart in Figure 1, between March 2006 and October 2016, a total of 257 female patients with early-stage TNBC were eligible for inclusion in this study. A total of 75 patients were excluded due to a lack of complete information, i.e., 38 patients did not have complete histological grade data, 30 patients did not have Ki-67 index data, 5 patients lacked data on haematological parameters, and 2 patients had missing T stage information.

The baseline clinicopathological features are shown in Table 1. The median age at the diagnosis of all eligible patients was 48.0 (IQR: 41.0–57.0) years old, and 150 (58.4%) patients were ≤50 years old. The median BMI was 23.4 (IQR: 23.0–23.8) kg/m^2^, and 132 (51.6%) patients had a value of BMI ≤23.0 kg/m^2^. There were 168 (65.4%) patients in the premenopausal period and 197 (76.7%) with a Ki-67 index value ≥30%. According to the seventh AJCC staging criteria, stages I, II, and III accounted for 23.0, 55.6, and 21.4%, respectively.

**TABLE 1:** Characteristics of patients eligible in this study.

Characteristics	All (N = 257)	IronMLRscore		*p* value
		Low (N = 228)	High (N = 29)	
Age (years), median (IQR)	48.0 (41.0–57.0)	48.7 (47.3–50.2)	50.7 (46.2–55.1)	0.406
Age at diagnosis				0.332
≤50	150 (58.4%)	136 (59.6%)	14 (48.3%)	
>50	107 (41.6%)	92 (40.4%)	15 (51.7%)	
BMI, median (IQR)	23.4 (23.0–23.8)	23.5 (23.1–23.9)	22.3 (21.2–23.6)	0.064
BMI				0.029
≤23	132 (51.6%)	111 (48.9%)	21 (72.4%)	
>23	124 (48.4%)	116 (51.5%)	8 (27.6%)	
T stage^a^				0.232
T1	89 (34.6%)	78 (34.2%)	11 (37.9%)	
T2	141 (54.9%)	125 (54.8%)	16 (55.2%)	
T3	23 (8.9%)	21 (9.2%)	2 (6.9%)	
T4	4 (1.6%)	4 (1.8%)	0 (0.0%)	
N stage^a^				0.403
N0	152 (59.2%)	132 (57.9%)	20 (69.1%)	
N1	57 (22.2%)	54 (23.7%)	3 (10.3%)	
N2	24 (9.3%)	21 (9.2%)	3 (10.3%)	
N3	24 (9.3%)	21 (9.2%)	3 (10.3%)	
Stage^a^				0.986
I	59 (23.0%)	52 (22.8%)	7 (24.1%)	
II	143 (55.6%)	127 (55.7%)	16 (55.2%)	
III	55 (21.4%)	49 (21.5%)	6 (20.7%)	
Menstrual status				0.546
Premenopausal	168 (65.4%)	151 (66.2%)	17 (58.6%)	
Postmenopausal	89 (34.6%)	77 (33.8%)	12 (41.4%)	
Pathological grade^b^				0.999
1/2	112 (43.6%)	99 (43.4%)	13 (44.8%)	
3	145 (56.4%)	129 (56.6%)	16 (55.2%)	
KI-67 index^c^				0.028
<30%	60 (23.3%)	48 (21.1%)	12 (41.4%)	
≥30%	197 (76.7%)	180 (78.9%)	17 (58.6%)	
Lymphovascular invasion				0.502
No	205 (79.8%)	180 (78.9%)	25 (86.2%)	
Yes	52 (20.2%)	48 (21.1%)	4 (13.8%)	

a Diagnosed based on the AJCC 2010 criteria (seventh edition).

b Histological grade at diagnosis was based on the degree of histological tumor differentiation.

c The Ki-67 index at diagnosis indicates DNA synthetic activity as measured using immunocytochemistry.

d The cut-off value was determined by means of maximally selected log-rank statistics.

Abbreviation: IQR, interquartile ranges.

**FIGURE 2:**
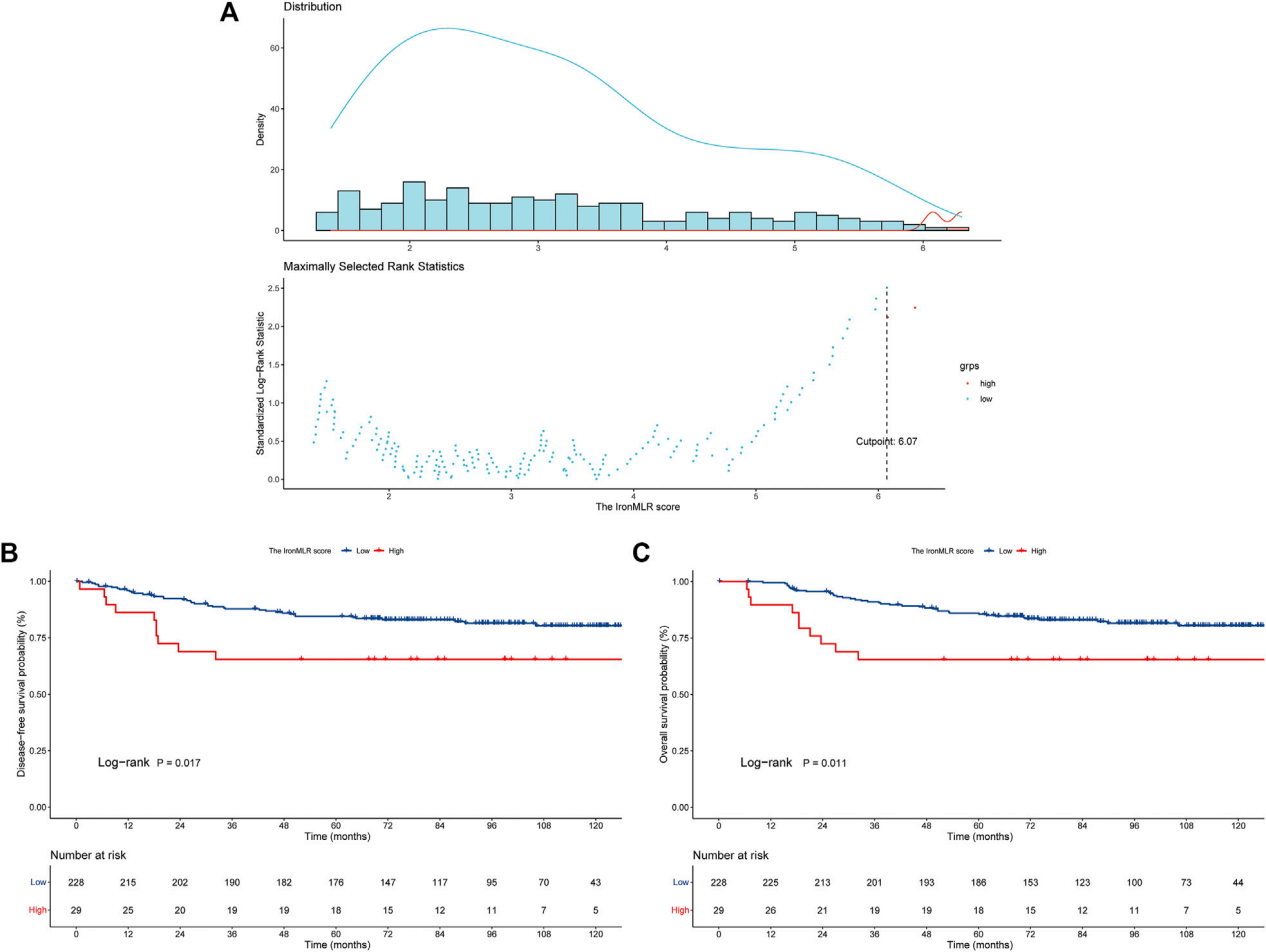
Determination of the cutoff value of the IronMLR score and Survival risk stratification of patients. **(A)** A cutoff value of 6.07 μmol/L defined by maximally selected rank statistics. **(B)** Survival curves for disease-free survival (DFS) between different IronMLR score groups. **(C)** Survival curves for overall survival (OS) between different IronMLR score groups.

The optimal cut-off value of the IronMLR score defined by the maximally selected rank statistics for OS was 6.07 umol/L (Figure 2A). Overall, 228 (88.7%) patients had a low IronMLR score, and 29 (11.3%) patients had a high IronMLR score. The baseline clinicopathological characteristics between these two IronMLR groups were much more balanced (Table 1). Compared to TNBC patients in the low IronMLR score group, a higher proportion of patients in the high IronMLR score group had a BMI ≤23 kg/m^2^ (*p* = 0.029) and Ki-67 index <30% (*p* = 0.028).

### Survival Outcomes

The median follow-up time was 92.3 months (95% CI 76.0–119.3 months). Compared to patients in the low IronMLR score group, early-stage TNBC patients in the high IronMLR score group had a significantly worse 5-years DFS rate (81.2%, 95% CI 76.2%–86.5% *vs.* 65.5%, 95% CI 50.3%–85.3%, *p* = 0.012) (Figure 2B) and 5-years OS rate (86.0%, 95% CI 81.6%–90.7% *vs.* 65.5%, 95% CI 50.3%–85.3%, *p* = 0.011) (Figure 2C).

### Independent Indicators for Survival

As shown in Table 2, five factors were considered independent indicators for DFS according to the univariate Cox regression model, including age, lymphovascular invasion, T stage, N stage, and the IronMLR score. Further multivariate Cox analysis demonstrated that N stage and the IronMLR score remained independent predictors of DFS for patients with early-stage TNBC.

**Table 2:** Univariate and multivariate cox regression analysis of disease-free survival.

Characteristics	Univariate cox analysis		Multivariate cox analysis	
	Hazard ratio (95%CI)	*p* value	Hazard ratio (95%CI)	*p* value
Age (year)				
≤50	Reference		Reference	
>50	1.723 (1.056–2.810)	0.029*	1.370 (0.810–2.310)	0.243
BMI				
≤23	Reference			
>23	0.946 (0.581-1.540)	0.823		
Menstrual status				
Premenopausal	Reference			
Postmenopausal	1.601 (0.974–2.632)	0.064		
Histological grade^a^				
1/2	Reference			
3	0.988 (0.605–1.613)	0.961		
Lymphovascular invasion				
No	Reference		Reference	
Yes	2.324 (1.358–3.979)	0.002*	1.560 (0.840–2.880)	0.157
Ki-67 index at diagnosis < 30%^b^				
No	Reference			
Yes	1.135 (0.655–1.964)	0.652		
T stage^c^				
1	Reference		Reference	
2	0.863 (0.506–1.474)	0.590	0.840 (0.490–1.460)	0.543
3	1.021 (0.415–2.508)	0.964	0.890 (0.350–2.250)	0.802
4	3.886 (1.165–12.970)	0.027*	2.660 (0.770–9.270)	0.123
N stage^c^				
0	Reference		Reference	
1	2.452 (1.325–4.538)	0.004*	2.360 (1.250–4.430)	0.008*
2	2.898 (1.381–6.083)	0.005*	2.560 (1.190–5.500)	0.016*
3	5.788 (2.925–11.453)	<0.001*	4.130 (1.910–8.920)	<0.001*
IronMLR score				
Low	Reference		Reference	
High	2.157 (1.171–3.974)	0.014*	2.310 (1.200–4.430)	0.012*

*p＜0.05.

a Histological grade at diagnosis was based on the degree of histological tumor differentiation.

b The Ki-67, index at diagnosis indicates DNA, synthetic activity as measured using immunocytochemistry.

c Diagnosed based on the AJCC, 2010 criteria (seventh edition).

Table 3 presents that age, menstrual status, lymphovascular invasion, T stage, N stage, and the IronMLR score of patients achieved the predominate threshold of OS (*p* < 0.05) for early-stage TNBC female patients in the univariate Cox analysis. Then, these variables were further analysed in the multivariate Cox regression model, which revealed that T stage, N stage, and the IronMLR score continued to be significantly associated with OS.

**TABLE 3:** Univariate and multivariate cox regression analysis of overall survival.

Characteristics	Univariate cox analysis		Multivariate cox analysis	
	Hazard ratio (95%CI)	*p* value	Hazard ratio (95%CI)	*p* value
Age (year)				
≤50	Reference		Reference	
>50	2.103 (1.199–3.689)	0.010*	1.770 (0.640–4.900)	0.272
BMI				
≤23	Reference			
>23	0.810 (0.463–1.416)	0.460		
Menstrual status				
Premenopausal	Reference		Reference	
Postmenopausal	1.859 (1.067–3.238)	0.029*	0.910 (0.320–2.570)	0.852
Histological grade^a^				
1/2	Reference			
3	1.235 (0.701–2.176)	0.464		
Lymphovascular invasion				
No	Reference		Reference	
Yes	2.689 (1.504–4.809)	0.001*	1.820 (0.920–3.600)	0.086
Ki-67 index at diagnosis < 30%^b^				
No	Reference			
Yes	0.891 (0.456–1.740)	0.735		
T stage^c^				
1	Reference		Reference	
2	0.880 (0.473–1.638)	0.686	0.820 (0.430–1.540)	0.535
3	1.363 (0.537–3.458)	0.514	1.020 (0.370–2.870)	0.963
4	5.251 (1.536–17.958)	0.008*	3.880 (1.070–13.990)	0.039*
N stage^c^				
0	Reference		Reference	
1	2.099 (1.002–4.397)	0.049*	1.990 (0.930–4.280)	0.077
2	3.640 (1.621–8.174)	0.002*	2.990 (1.260–7.060)	0.013*
3	7.134 (3.392–15.006)	<0.001*	4.510 (1.940–10.500)	<0.001*
IronMLR score				
Low	Reference		Reference	
High	2.404 (1.201–4.808)	0.013*	2.730 (1.310–5.710)	0.008*

*p＜0.05.

a Histological grade at diagnosis was based on the degree of histological tumor differentiation.

b The Ki-67 index at diagnosis indicates DNA synthetic activity as measured using immunocytochemistry.

c Diagnosed based on the AJCC 2010 criteria (seventh edition).

### Establishment and Evaluation of the Prognostic Model

**FIGURE 3:**
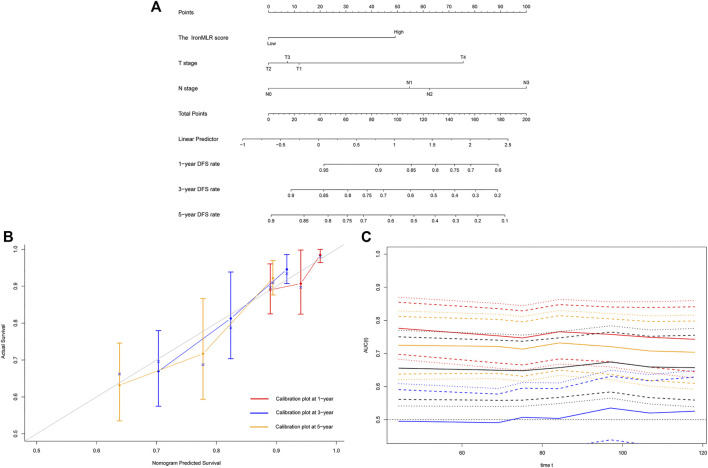
Development and evaluation of a model for individualized prediction of disease-free survival (DFS). **(A)** Nomogram of prognostic model for patients with early-stage triple-negative breast cancer. **(B)** Calibration plots of 1-, 3-, and 5-years DFS predictions. **(C)** Time-dependent receiver operating characteristic (ROC) curves (Nomogram [red], T stage [blue], N stage [orange], TNM stage [black]).

Based on independent indicators for DFS identified in the above multivariate Cox analysis, i.e., N stage and the IronMLR score, we established a prognostic model for individualized DFS prediction for patients with early-stage TNBC. Given that T stage is commonly used in clinical practice, we also integrated it into the prognostic model (Figure 3A). The model showed perfect predictive performance with a good C-index of 0.725 (95% CI 0.662–0.788). Satisfactory consistency between observational 1-, 3-, and 5-years DFS rates and nomogram-predicted DFS rates was found in calibration curves (Figure 3B). Time-dependent ROC curves showed that the area under the curve (AUC) of our nomogram for DFS was higher than the AUCs for T stage, N stage, and the traditional tumor-node-metastasis (TNM) staging system (Figure 3C).

**FIGURE 4:**
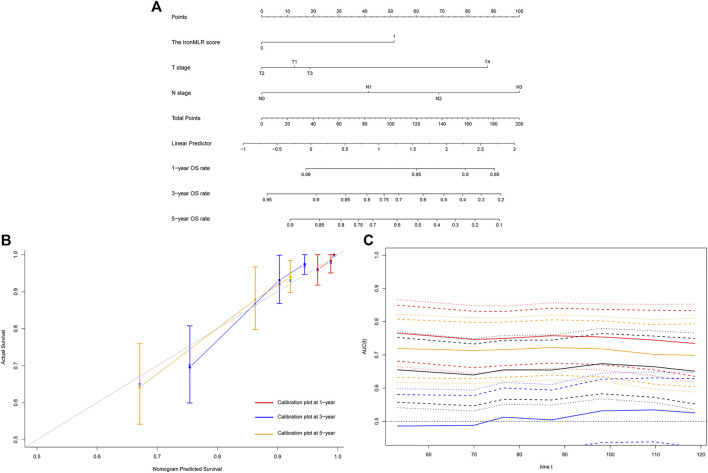
Development and evaluation of a model for individualized prediction of overall survival (OS). **(A)** Nomogram of prognostic model for patients with early-stage triple-negative breast cancer. **(B)** Calibration plots for predicting OS at 1-, 3-, and 5-years. **(C)** Time-dependent receiver operating characteristic (ROC) curves (Nomogram [red], T stage [blue], N stage [orange], TNM stage [black]).

Similarly, we developed a prognostic model with independent indicators according to the multivariate Cox regression model, i.e., T stage, N stage, and IronMLR score, for individualized OS prediction (Figure 4A). The predictive accuracy of our nomogram for OS was very good, with a satisfactory C-index of 0.758 (95% CI 0.689–0.826). The calibration plots for 1-, 3-, and 5-years OS rates demonstrated good consistency between actual and nomogram-predicted OS rates (Figure 4B). Compared to traditional T stage, N stage, and TNM stage, our prognostic nomogram for OS achieved a higher AUC according to time-dependent ROC curves (Figure 4C).

## Discussion

In this study, we constructed a novel comprehensive biomarker based on the serum iron level and systemic inflammation status, the IronMLR score. According to a cut-off value of 6.07 determined by maximally selected rank statistics, heterogeneous patients with early-stage TNBC were divided into groups based on a low or high IronMLR score. Significant differences in DFS and OS were found between these two groups. Further univariate and multivariate Cox regression analyses demonstrated that a high IronMLR score was a negatively independent predictor of poor survival for early-stage TNBC patients. Subsequently, prognostic models integrating the IronMLR score and two clinicopathological features (T stage and N stage) for DFS and OS were established and graphically depicted as nomograms. These prognostic nomograms presented good discriminative ability and satisfactory predictive agreement between observed clinical outcomes and the nomogram-predicted survival probability.

Although iron is indispensable for many proteins and enzymes, playing an important role in various biological processes (Hider and Maret, 2015; Torti et al., 2018), it also attributes to oxidative stress and DNA damage. Excess iron has been found to be significantly associated with tumor initiation, progression, aggressiveness, and metastasis (Chen et al., 2015; Guo et al., 2015; Radulescu et al., 2016). Increasing studies have found iron metabolic dysregulation, iron homeostatic disorder, and distribution changes in peripheral iron in patients with various malignancies, including breast cancer (Torti et al., 2018; Galaris et al., 2019). Meanwhile, iron accumulation could induce lipid peroxidation and facilitate lethal injury to tumor cells, which subsequently could contribute to inhibiting tumor initiation and progression (Chang et al., 2019; Forciniti et al., 2020; Yang et al., 2021). Iron accumulation is involved in some programmed cell death pathways, such as apoptosis, necroptosis, ferroptosis, and so on (Dixon et al., 2012; Torti et al., 2018). Thus, iron-related metabolism or iron chelators might be potential novel therapeutic targets and strategies for antitumor treatment (Nutting et al., 2009; Yamasaki et al., 2011; List et al., 2012; Neufeld et al., 2012; Kalinowski et al., 2016). Hence, the measurement of iron levels is necessary. Many previous studies have used iron-bound proteins such as transferrin and ferritin to reflect body iron levels (Fonseca-Nunes et al., 2014; Torti et al., 2018; Morales and Xue, 2021), but this indirect test might produce errors in reflecting actual iron levels, direct detection of serum iron would be better (Torti et al., 2018; El Hout et al., 2018). Thus, we adopted the peripheral iron level to construct the IronMLR score in this study.

Inflammation, a hallmark feature of tumors, has shown a significant association with tumorigenesis, progression, development, and metastasis (Diakos, et al., 2014). Cancer-associated inflammation refers to complicated connections between tumors and inflammatory responses, and it might show a significant relation to poor prognosis and therapeutic failure (Zitvogel et al., 2017; Li et al., 2021). As vital inflammatory mediators, peripheral inflammatory cells or inflammatory cells infiltrating in the TME are potential prognostic biomarkers for breast cancer (Diakos et al., 2014; van der Willik et al., 2018). Among inflammatory cells, monocytes have been identified as inhibitors of lymphocyte activation and play an important role in tumor aggressiveness and metastasis (Tiainen et al., 2021). Circulating lymphocytes or lymphocytes infiltrating in the TME are both involved in antitumor immune responses (Andre et al., 2013). Therefore, the circulating marker MLR might reflect systemic inflammatory status, an elevated MLR indicates an increased monocyte count or a reduced lymphocyte count, which suggests poor antitumor immunity (Miklikova et al., 2020). The negative association between the MLR and survival outcomes of breast cancer patients has also been explored (De Giorgi et al., 2019).

Nowadays, the underlying association between iron and inflammation is still poorly understood. As previously mentioned, ferroptosis plays multifaceted functions in cancer-related inflammation, activation, and infiltration of inflammatory cells (Umemura et al., 2017; Tsurusaki et al., 2019; Sun et al., 2020; Zhou et al., 2020). In addition, IL-6, an inflammatory cytokine, is secreted locally in the TME of breast cancer such as monocytes (Masjedi et al., 2018), and it has been demonstrated to directly regulate systemic iron homeostasis through IL6/IL6R/Janus kinase 2 (JAK2)/signal transducer and activator of transcription 3 (STAT3) signaling pathway (Hentze et al., 2010; Ganz and Nemeth, 2011; Guo et al., 2015). Moreover, IL-6 also likely contributes to the dysregulated hepcidin/ferroportin signaling in breast cancer and mediates further iron homeostasis (Masjedi et al., 2018). So, the blockade of IL-6 inflammatory signaling cascade has been investigated as a potential therapeutic strategy to suppress hepcidin in breast cancer and for anaemic cancer patients (Jiang et al., 2011; Guo et al., 2015). In recent years, researchers have taken great interest in developing prognostic models for risk stratification and clinical outcomes predictions. Predictive models incorporating multiple biomarkers rather than a single marker have higher prognostic accuracy (Li et al., 2021). Thus, we comprehensively developed a new biomarker integrating serum iron with systemic inflammatory markers. Our analyses showed that the novel IronMLR score is an independent indicator of the survival of patients with early-stage TNBC, and patients stratified according to its cut-off value experience significantly different survival outcomes.

Based on the classification of the IronMLR score, prognostic nomogram incorporating it and the commonly used T stage and N stage were established. In clinical practice, 21-gene tests, 70-gene assays, and PAM50 predictive models are widely applied for risk stratification or therapeutic recommendations, but these genetic models are restricted to patients with specific subtypes, lymph node-negative patients or women at high clinical risk of recurrence from breast cancer, with limited prognostic accuracy (Dixon et al., 2012; Gnant et al., 2015; Ibraheem et al., 2020; Poorvu et al., 2020). Comparatively speaking, the predictive nomogram developed in our study were more accurate with a high C-index, economic, convenient, and easier to be applied in primary hospitals. Clinicians and patients usually use the TNM staging system to stratify recurrence or death risks and to guide therapeutic strategies, however, the TNM criteria just incorporate a limited number of clinical features, so its predictive accuracy is limited due to the heterogeneity observed among patients (Bareche et al., 2018; Grosselin et al., 2019). Our time-dependent ROC curves showed that compared to the common TNM staging system, higher AUCs for DFS and OS were achieved with our prognostic nomograms, which suggested that our predictive models have greater predictive accuracy and might be a strong supplement to traditional TNM criteria. For example, TNBC patients with high IronMLR scores tended to have poorer prognosis, for them, more intensive care, closer follow-up, and more precise routine imaging monitor such as CT or magnetic resonance imaging (MRI) instead of breast/abdominal ultrasound would be much meaningful to monitor their tumor condition and improve their survival outcomes as much as possible.

Our study had several limitations, which should be acknowledged. First, because we retrospectively explored the prognostic value of the IronMLR score, selection bias is inevitable. Second, we only evaluated the baseline serum iron level and MLR before the initiation of any anti-tumor therapy, but we failed to explore the dynamic changes of the serum iron level and inflammatory status of patients during subsequent treatment. Monitoring changes in these parameters might help clinicians both to adjust the therapeutic strategy in time and to guide personalized treatment. Third, this study was a preliminary research, underlying mechanisms of the IronMLR on TNBC, and the relationship between MLR and iron require more investigations. Finally, patients with early-stage TNBC included in this study were from only a single centre in China. Further large or multicentre cohort studies are warrant to enhance the power of our results. Although we actively sought help and cooperation from other hospitals to validate our prognostic models, unfortunately, we failed to obtain complete haematological parameters and follow-up information. Thus, the prognostic accuracy of our nomograms for early-stage TNBC patients from other geographic backgrounds requires further evaluation in future studies.

## Conclusion

In conclusion, we propose the Iron-inflammation axis, the IronMLR score, as a novel prognostic biomarker for trace element and systemic inflammatory status in female patients with early-stage TNBC. Prognostic models based on the IronMLR score for individualized survival predictions showed good predictive performance and discriminative accuracy, but validation of the predictive role of the IronMLR score is needed in a large or multicentre cohort. Additional studies, especially those exploring the IronMLR score as a potential dynamic biomarker related to survival outcomes during treatment, are warranted in future.

## Data Availability

The raw data supporting the conclusion of this article will be made available by the authors, without undue reservation.
